# Regional variability of cardiovascular magnetic resonance access and utilization in the United States

**DOI:** 10.1016/j.jocmr.2024.101061

**Published:** 2024-07-11

**Authors:** Jennifer M. Li, David R. Ho, Nazia Husain, Robert W. Biederman, J. Paul Finn, Anthon R. Fuisz, Ibrahim M. Saeed, Kim-Lien Nguyen

**Affiliations:** a University of Arizona College of Medicine, Phoenix, Arizona, USA; b David Geffen School of Medicine at UCLA, Los Angeles, California, USA; c Lurie Children’s Hospital and Northwestern University School of Medicine, Chicago, Illinois, USA; d West Virginia University, Morgantown, West Virginia, USA; e Carnegie Mellon University, Pittsburgh, Pennsylvania, USA; f Medical University of South Carolina and Roper St Francis Hospital, Charleston, South Carolina, USA; g Westchester Medical Center, Valhalla, New York, USA; h Virginia Heart, Falls Church, Virginia, USA; i Inova Schar Heart and Vascular, Fairfax, Virginia, USA; j VA Greater Los Angeles Healthcare System, Los Angeles, California, USA

**Keywords:** Cardiovascular magnetic resonance imaging, Utilization, Imaging access, Geographic density, Advocacy, Cost-effectiveness

## Abstract

**Background:**

Clinical guidelines and scientific data increasingly support the appropriate use of cardiovascular magnetic resonance (CMR) . The extent of CMR adoption across the United States (US) remains unclear. This observational analysis aims to capture CMR practice patterns in the US.

**Methods:**

Commissioned reports from the Society for Cardiovascular Magnetic Resonance (SCMR), pre-existing survey data from CMR centers, and socioeconomic and coronary heart disease data from the Centers for Disease Control and Prevention were used. The location of imaging centers performing CMR was based on 2018 Medicare claims. Secondary analysis was performed on center-specific survey data from 2017–2019, which were collected by members of the SCMR US Advocacy Subcommittee for quality improvement purposes. The correlation between the number of imaging centers billing for CMR services per million persons, socioeconomic determinants, and coronary heart disease epidemiology was determined.

**Results:**

A total of 591 imaging centers billed the Center for Medicare & Medicaid Services for CMR services in 2018 and 112 (of 155) unique CMR centers responded to the survey. In 2018, CMR services were available in almost all 50 states. Minnesota was the state with the highest number of CMR centers per million Medicare beneficiaries (52.6 centers per million), and Maine had the lowest (4.4 per million). The total density of CMR centers was 16 per million for US Medicare beneficiaries. Sixty-eight percent (83 of 112) of survey responders were cardiologists, and 28% (31/112) were radiologists. In 72% (71/112) of centers, academic health care systems performed 81%–100% of CMR exams. The number of high-volume centers (>500 scans per year) increased by seven between 2017 and 2019. In 2019, 53% (59/112) of centers were considered high-volume centers and had an average of 19 years of experience. Centers performing <50 scans had on average 3.5 years of experience. Approximate patient wait time for a CMR exam was 2 weeks to 1 month.

**Conclusion:**

Despite increasing volume and availability in almost all 50 states, CMR access remains geographically variable. Advocacy efforts to improve access and innovations that reduce imaging time and exam complexity have the potential to increase the adoption of CMR technology.

## Graphical abstract

**Figure:**
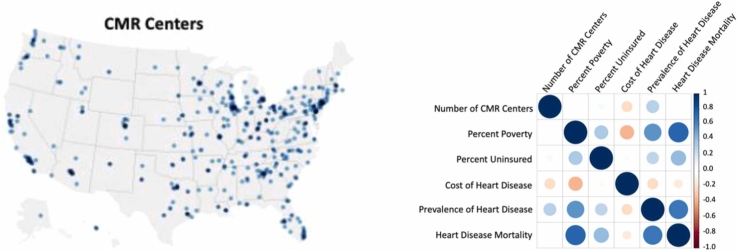
Billing data from the Centers for Medicare & Medicaid Services along with survey data were used to evaluate the adoption of cardiovascular magnetic resonance (CMR) technology in the United States. Despite increasing CMR imaging volume and availability in almost all 50 states, access to CMR services remains geographically variable. Advocacy efforts to improve access and innovations that reduce imaging time and exam complexity have the potential to increase adoption of CMR technology.

## 1 Introduction

Cardiovascular disease continues to be the leading cause of death for Americans over 65 years old [1] with more than 40% of Medicare beneficiaries reported being affected by at least one heart condition [2]. To address the growing burden of cardiovascular disease, emerging technology for the prevention, diagnosis, and treatment of cardiovascular disease has been integrated into current clinical guidelines. In parallel, cardiovascular imaging has become increasingly recognized as a vital tool in early disease characterization, risk stratification, and treatment guidance partly due to an overall shift of health care toward precision medicine [3], [4] and prevention.

Of the various imaging modalities, cardiovascular magnetic resonance (CMR) imaging is considered the reference standard for many applications, including cardiac function through assessment of morphology, ejection fraction, volume, and myocardial fibrosis [5], [6]. Recent guidelines specify other indications for CMR including (1) ischemic heart disease [7], [8], (2) congenital heart disease [9], and (3) heart failure [10]. Indeed, stress CMR is now recommended for the assessment of coronary artery disease (CAD) by both the American College of Cardiology (ACC)/American Heart Association (AHA) [7] and ESC guidelines. [8] Despite these indications and guideline recommendations, CMR remains underutilized in the United States (US), making up less than 1% of all noninvasive cardiac imaging performed from 2011 to 2015 [11]. According to data from the Organization for Economic Co-operation and Development, the US was only second to Japan in the number of magnetic resonance imaging (MRI) units per million inhabitants [12]. The US had 40.4 MRI units per million in 2019 and Japan had 57.3 in 2020. Despite cardiovascular disease being the number one cause of mortality and morbidity, cardiac MRI exams only constituted 1% while vascular magnetic resonance angiography constituted 5% of all exams in 2015 [13]. When compared with guidelines from the European Society of Cardiology, CMR is less represented in guidelines produced by the AHA and ACC, suggesting that CMR might be better supported and implemented in countries outside of the US [14].

Given disparities in guideline recommendations and observations in clinical practice, the objective of this work is to determine the extent of clinical CMR adoption in the US based on Medicare data derived from commissioned administrative reports and survey data from CMR centers.

## 2 Methods

Institutional review board approval was not required for the secondary analysis of pre-existing survey data, which were collected for administrative and quality improvement purposes. Institutional review board approval was also not required for the analysis of publicly available data.

### 2.1 Data sources

We conducted a retrospective cross-sectional analysis using administrative data obtained by The Moran Company, a health care research and consulting firm. These data were commissioned by the Society for Cardiovascular Magnetic Resonance (SCMR) and were based on Medicare physician billing practices for 2018 in accordance with data use agreements set forth by the Center for Medicare & Medicaid Services (CMS). The data were from the 2018 hospital outpatient prospective payment system. The specific number of claims from sites with 10 or fewer claims was omitted. Centers included in the report billed for at least one CMR study in 2018. The billing codes from the Healthcare Common Procedure Coding System (HCPCS) included cardiac MRI for morphology (75557), cardiac MRI with stress imaging (75559), cardiac MRI for morphology with and without contrast (75561), and cardiac MRI with stress imaging with and without contrast (75563). These data were used to map the geographic availability of CMR centers. We obtained 2018 census data for Traditional Medicare enrollees from the Kaiser Family Foundation (https://KFF.org), an independent non-profit health policy research organization, and computed the number of CMR centers per million Medicare beneficiaries [15].

### 2.2 Electronic survey data

Survey data were collected for the purpose of quality improvement by members of the SCMR US Advocacy Subcommittee. The analysis performed in this study represents the secondary analysis of pre-existing data. Briefly, a contact list consisting of 591 unique centers based on the Moran report was generated. To identify the physician champion of each facility, the title “director of CMR” or “director of advanced cardiovascular imaging” was used, or when not available, the title “director of cardiac imaging” was used in an online search of the facility’s website. Based on centers with contact information available online, an electronic survey was sent (Google Forms, Google, Mountain View, California) to a final list of 155 centers. The survey was also posted on the 2023 SCMR Scientific Sessions conference mobile application and distributed through the SCMR email list server. The survey contained 42 questions and was designed to be completed within 15 minutes. A complete list of survey questions is provided in Supplemental Material Table S1.

### 2.3 Analysis

Data are summarized and reported as means or medians and percentages. Where appropriate, the range is also provided. CMR centers based on CMS data are displayed as geomaps and the number of CMR centers per million persons are represented as color overlays. To illustrate the geographic variability of CMR centers across the US, maps depicting CMR center distribution/density were created. To provide a visual comparison of geographic access to CMR centers relative to several measures of socioeconomic determinants (Area Deprivation Index, poverty, health insurance) and coronary heart disease outcomes (prevalence, death rates), we obtained publicly available data from the University of Wisconsin-Madison (Area Deprivation Index) [16], [17] and the Centers for Disease Control and Prevention (CDC) [18] and downloaded the data as color maps. We used the Mapping Function from the University of Wisconsin-Madison Neighborhood Atlas to generate a color map of the 2020 distribution of Area Deprivation Index, which reflected socioeconomic disadvantage percentiles for US zip codes based on 17 US Census-based metrics. Correlation between the number of imaging centers billing CMS for 2018 CMR services per state and CDC-reported measures of socioeconomic determinants (percent poverty, percent uninsured, cost of heart disease) and coronary heart disease epidemiology (heart disease prevalence, heart disease mortality) was computed (R, version 4.3.1, R Foundation for Statistical Computing, Vienna, Austria). Cost and prevalence data for heart disease in Medicare beneficiaries were provided by the county and combined for total values by state.

## 3 Results

The Moran report provided data on 591 unique centers in the US that billed CMS for at least one CMR service in 2018. According to data from the Kaiser Family Foundation, there were 37.99 million Traditional Medicare beneficiaries in 2018. The total density of imaging centers that billed CMS for CMR services in 2018 was 15.6 centers per million US Medicare beneficiaries. A total of 112 centers responded to the survey. Of the 112 unique physician champions (each representing one unique CMR center) included in the survey, 78 (70%) were cardiologists and 31 (28%) were radiologists. Twenty (18%) centers had three to five CMR trainees. Eight (7%) had greater than five trainees, while 45 (40%) had no trainees. Most surveyed physicians answered “somewhat agree” to the question “Do you feel supported by your institution to read CMRs from a time perspective?” while 15 answered “definitely disagree.” In response to the same question from a salary perspective, a total of 24 answered “definitely disagree,” while 27 answered “definitely agree.”

### 3.1 Geographic distribution of CMR centers

**Fig. 1:**
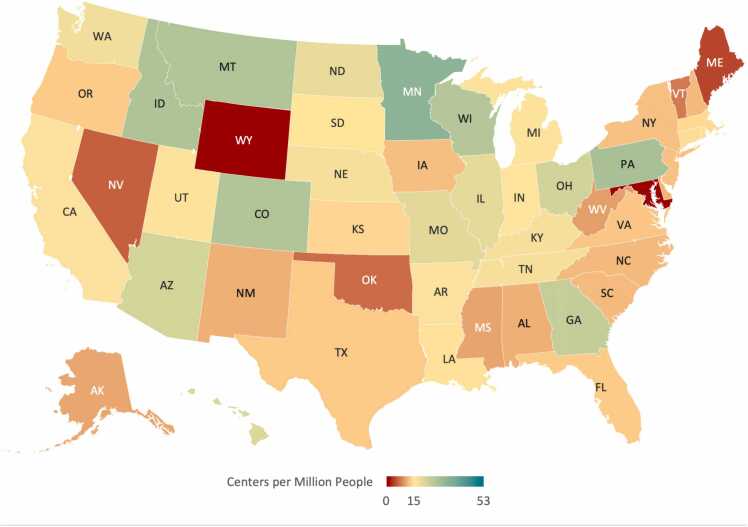
Distribution of imaging centers that billed Medicare for cardiovascular magnetic resonance services 2018 (N = 562). Data are reported as centers per million persons (50th percentile = 15) using 2018 Traditional Medicare enrollment census data.

Fig. 1 summarizes CMR centers per million Medicare beneficiaries in the US. For every million Medicare beneficiaries in the US in 2018, there were 16 centers that billed Medicare for CMR services. In 2018, two states (Maryland and Wyoming) did not have any CMR claims listed, which may be due to a different payer arrangement. CMR center distribution was most concentrated along the East Coast and Midwest, with some areas on the West Coast having higher CMR center density (California). The number of centers providing CMR per million persons was highest in the District of Columbia, Minnesota, and Pennsylvania where the ratios were 52.6, 33.5, and 28.7, respectively. Excluding states that did not bill Medicare for CMR services, Maine, Nevada, and Oklahoma had the lowest ratios of imaging centers that billed Medicare for CMR services per million beneficiaries at 4.4, 6.1, and 6.9, respectively.

**Fig. 2:**
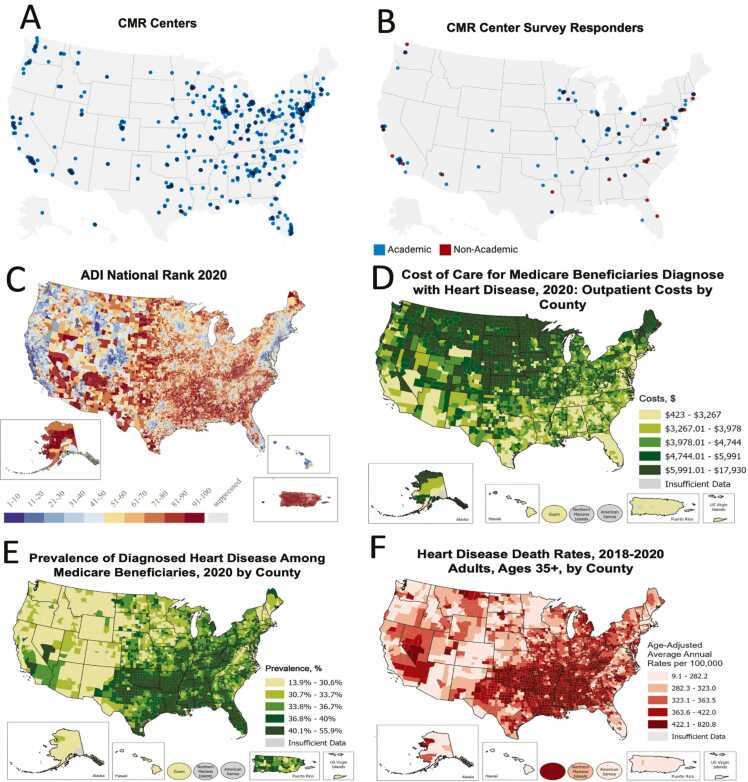
Comparison of centers billing Medicare for CMR services, CMR center survey responders, socioeconomic distribution, and coronary heart disease. Shown are color map distribution of imaging centers that billed Medicare for cardiovascular magnetic resonance services in 2018 (A, n = 562), CMR center survey responders (B, n = 112), socioeconomic determinants (C), cost of outpatient care (D), and coronary heart disease (E and F). For panels A and B, data are reported per zip code. Each blue dot represents one CMR center. For panel B, academic centers are defined as centers that self-reported performing >50% of their scans in an academic health care system. The 2020 Area Deprivation Index (ADI) color map [16], [17] shows socioeconomic disadvantage percentiles for US zip codes based on 17 US Census-based metrics (C). A block group with a ranking of 1 (bluish) indicates the lowest level of “disadvantage” and an ADI ranking of 100 (reddish-orange) indicates the highest level of “disadvantage”. Based on the CDC color maps, [17] regions with the highest outpatient cost of care for heart disease (D) and the highest prevalence of heart disease (E) in Medicare beneficiaries are color-coded in dark green. Regions with the highest heart disease death rates (age >35 years, 2018–2020) by county are coded as dark red (F). *CDC* Centers for Disease Control and Prevention, *CMR* cardiovascular magnetic resonance.

**Fig. 3:**
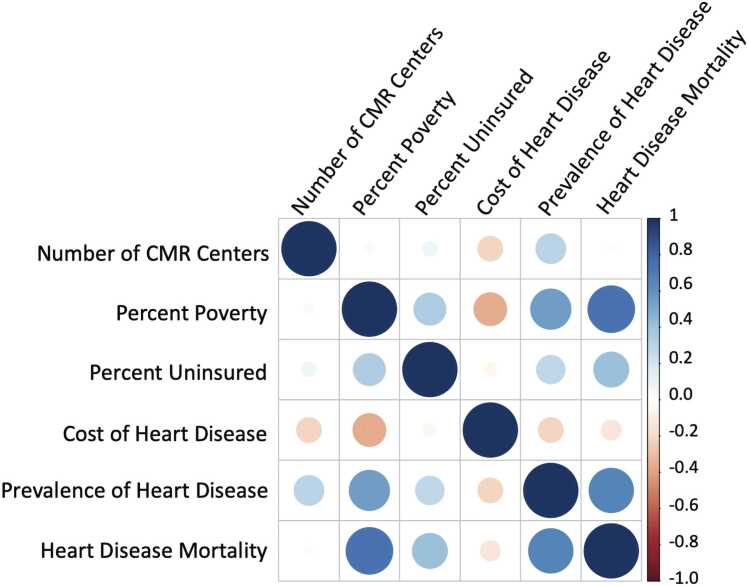
Correlation matrix between the number of imaging centers billing CMS for 2018 CMR services per state and CDC-reported measures of socioeconomic determinants and coronary heart disease epidemiology [17]. p-value was significant for the correlation between number of CMR centers and prevalence of heart disease (p = 0.03) “Number of centers” = number of centers billing CMS in 2018 for CMR services per state, “Percent Poverty” = percent of population living in poverty (2018), “Percent Uninsured” = percent of population without health insurance (2018), “Cost Heart Disease” = cost of care for Medicare beneficiaries diagnosed with heart disease (2020), “Prevalence Heart Disease” = prevalence of diagnosed heart disease among Medicare beneficiaries (2020), “Heart Disease Mortality” = heart disease death rates among adults age 35+ (2018–2020). CDC data were downloaded from the Interactive Atlas of Heart Disease and Stroke (https://www.cdc.gov/dhdsp/maps/atlas/index.htm). *CDC* Centers for Disease Control and Prevention, *CMS* Centers for Medicare & Medicaid Services, *CMR* cardiovascular magnetic resonance.

Fig. 2 provides a comparison between the geographic distribution of CMR centers that billed Medicare, socioeconomic determinants, cost of outpatient care in Medicare beneficiaries with CAD, prevalence of diagnosed heart disease, and heart disease death rates. CMR centers were geographically more dense in areas of the lowest socioeconomic disadvantage (lowest Area Deprivation Index), low poverty, and lowest percentage of people lacking health insurance. Fig. 3 provides a correlation matrix of the number of centers relative to several CDC-measured statistics related to socioeconomic determinants and epidemiology of coronary heart disease. There was a non-significant inverse correlation between the number of CMR centers and the cost of care for Medicare beneficiaries with heart disease (r = −0.21, p = 0.14).

### 3.2 CMR center characteristics and volume

**Fig. 4:**
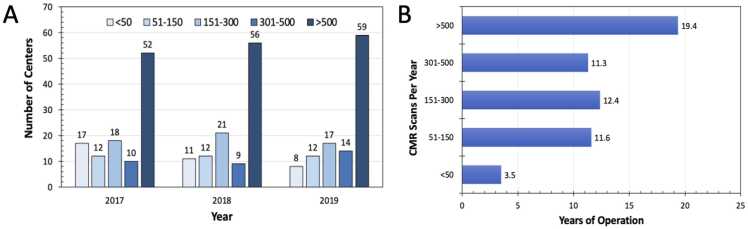
Number of cardiovascular imaging centers (A) and imaging volume (B) from 2017–2019 (N = 112) based on CMR center survey. *CMR* cardiovascular magnetic resonance.

Of the 112 survey responses, seventy-one centers indicated 81%–100% of their scans were performed in an academic health care system, while 21 centers reported 81%–100% of their scans were performed in a non-academic setting. Seventy-three (68%) centers reported 0%–20% of their scans were performed in an inpatient setting, while 54 (48%) reported 81%–100% of scans were performed in the outpatient setting. A higher proportion of CMR centers used lower magnet field strength (1.5T magnets: 63%, N = 60 centers; 3T magnets: 38%, N = 36 centers). Based on Medicare billing from the Moran data, cardiac MRI for morphology with contrast was the most frequently billed CMR procedure (range of <10–465). In 2019, 269 out of 567 (47%) centers billing for this procedure performed fewer than 10 of these studies. Of the 112 centers that responded to the survey, a total of 59 (53%) centers indicated that >500 scans were performed at their center in 2019 (Fig. 4A). From 2017–2019, the number of centers performing >500 scans annually increased from 52 to 59 centers, while the number of sites performing <50 scans decreased from 17 to 8. A center’s volume of scans per year is related to years in operation (Fig. 4B). Centers performing >500 scans per year in 2019 had an average of 19 years of experience, whereas centers with <50 scans per year had on average 3.5 years of experience. Sites performing between 51 and 150, 151 and 300, and 301 and 500 scans/year each had a similar average of 11–12 years of experience.

### 3.3 Wait time and operating hours of CMR centers

Based on data from 112 centers that responded to the SCMR survey, the wait time at 50 (45%) centers was 2 weeks to 1 month while at 37 (33%) centers, the wait time was 1–3 months. Only eight (7%) centers had a mean wait time >3 months. A total of 16 (14%) centers indicated they did not have a program in place to triage requests for CMR. At other centers, triaging was performed by only cardiologists (35%, N = 39), followed by only radiologists (17%, N = 19), or a combination of the two (9%, N = 10). Other options for triaging included cardiac technologists (10%, N = 11), registered nurses, and schedulers. At five (4%) centers, less than 6 hours/day of scan time was allotted to cardiovascular indications. At 33 (29%) centers, >10 hours/day were allocated to cardiovascular exams. Thirteen centers reported 1–3 days of operation per week, while 29 reported operating 6–7 days per week.

### 3.4 Indications

**Fig. 5:**
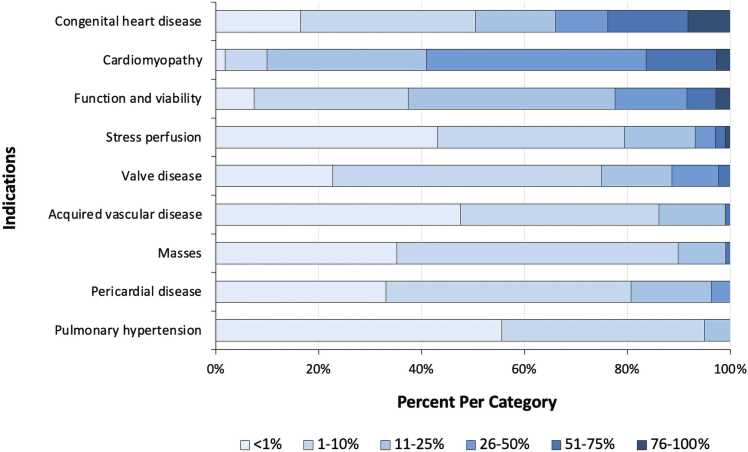
Indications for cardiovascular magnetic resonance exams. Percentages of responses were calculated for each category based on a center-specific survey.

Sixteen (14%) responders did not list an answer for every indication. Fig. 5 illustrates the proportion of responders for each frequency category by indication. These categories represent the percentage of CMR scans being performed for each specific indication out of the total number of CMR scans being performed at each institution. Based on survey responses, congenital heart disease was most often the majority indication at individual institutions. As a whole group, however, the overall most common indication was cardiomyopathy, followed by function and viability. The least frequent indications included stress perfusion and pulmonary hypertension.

## 4 Discussion

This study endeavored to determine the extent of clinical CMR adoption in the US The SCMR commissioned data provided information about the geographic location of CMR centers based on Medicare billing and an electronic survey provided data on center-specific CMR practice patterns. Based on these data, we found that CMR access remains geographically variable. There were 16 centers per million Medicare beneficiaries. CMR volume increased between 2017 and 2019 and the majority of CMR exams were performed at specialized, academic health care systems.

Despite a growing body of evidence and guidelines supporting the use of CMR, there continues to be wide variation in the geographic distribution of CMR centers, availability of CMR (based on wait time), and use of CMR (based on center-reported indications) in clinical practice. Although CMR imaging rates per 100,000 Medicare beneficiaries have risen by 125% [19], access to CMR remains geographically variable with few physicians and/or practices providing CMR services relative to echocardiography and nuclear imaging in each state [19].

Our results further confirm significant disparities in geographic access to CMR services despite cardiovascular disease being the leading cause of morbidity and mortality. CMR in the US is not equally accessible in all regions and mirrors global variation in CMR practice models [20]. In the US, areas with the highest density of CMR centers were along the East Coast. On the West Coast, CMR centers were more concentrated around large cities, such as Los Angeles. While greater density of heart disease burden and death rates are concentrated in the southeast, these areas do not show a greater density of CMR centers. Despite some states such as Montana and Pennsylvania having high ratios of CMR centers to population, CMR access may still be challenging due to long distances to the nearest CMR center. Prior studies have demonstrated that extremely disadvantaged US zip codes (identified by Area Deprivation Index scores) are less likely to have access to imaging facilities with MRI when compared to extremely advantaged areas (19% vs 32%) [21]. For rural areas and areas with significant poverty where additional factors, such as access to transportation, childcare, and health care insurance, are routine, the lack of CMR centers may further exacerbate existing health disparities. Although the inverse relationship between the number of CMR centers and health care expenditures for Medicare beneficiaries with heart disease was not statistically significant in this study, prior cost-effectiveness studies [22], [23], [24], [25] have shown the benefit of CMR for reducing downstream health care costs. We speculate that increased access to and utilization of CMR according to appropriate use criteria may play an important role in early diagnosis and potentially reduce overall health care spending.

Other barriers to CMR access and utilization include wait time (availability) and operating hours of CMR centers. In 2018, 47% (269/567) of centers performing the most billed procedure, cardiac morphology with contrast, billed for less than 10 scans. It is unclear how factors related to referral patterns, physician reader expertise, dedicated magnet time, physician compensation, or patient inaccessibility (insurance coverage, travel distance, etc) contribute to the high prevalence of low-volume centers. One possible explanation based on our survey data is a preference for referrals to larger academic centers; it remains unclear whether the preference for referral to larger academic centers is related to internal infrastructure (or overhead) for the performance of high-quality CMR exams.

The most common indication for CMR overall was the evaluation of cardiomyopathy, followed by congenital heart disease. While CMR offers a high-quality noninvasive method for visualizing these conditions, other cheaper modalities that require less technical overhead may be preferred due to financial and geographic constraints [26], [27]. It remains to be seen whether access to remote scanning capabilities, telehealth consultations, and emphasis on focused CMR exams will improve access and encourage more centers with magnetic resonance technology to provide CMR exams. Other innovations, such as faster imaging and push-button CMR, may also improve access and availability to CMR services.

Another barrier that merits discussion is physician and institutional compensation. A thorough historical perspective of cardiovascular imaging payment and reimbursement systems in the US is beyond the scope of the current work, and readers are referred to a paper by the Imaging Council of the ACC [28]. While many physicians indicated satisfaction with the level of financial support from their institution, almost as many indicated complete dissatisfaction. From 2012–2017, the average change per year in physician payment for inpatient CMR studies ranged from a $1.11 to $0.09 increase, depending on the type of study. For outpatient CMR studies, the average change per year in physician payment actually showed a decline, with a $54.56 decrease for stress CMR exams [19]. Many factors contribute to physician and institutional compensation and are beyond the scope of this analysis, but recent findings suggest that reimbursement arrangements for nuclear cardiac exams may have contributed to a higher proportion of the overall noninvasive cardiovascular imaging activity [11]. One pragmatic factor is the upkeep associated with magnetic resonance facilities. Low-volume centers may struggle to pay for their upkeep because magnet maintenance costs often surpass $10,000/month [29], and other study indications yielding higher reimbursement may receive priority booking. Given the extra time requirements for CMR exams and the need for dedicated technologists and physicians, additional CMR reimbursement may incentivize availability and access.

## 5 Limitations

First, this cross-sectional analysis is only applicable to the US and may not be reflective of other health care systems where physicians are salaried and reimbursement schemes are not considered fee-for-service. Our report is derived from a survey of physician leads at imaging centers providing CMR services and US Medicare billing data obtained through commissioned administrative reports. The latter source does not include centers that did not bill CMS. Specifically, Maryland has a state-wide fixed reimbursement amount for hospital procedures as well as National Institutes of Health-affiliated sites that did not bill for clinical exams. Another intrinsic limitation of CMS billing data is the lack of “real-time” or “up-to-date” availability, i.e. data are often several years old. However, our data are reflective of a pre-COVID-19 era and are likely to be more homogenous. Second, data used for the correlation analysis and geomap comparisons were from disparate years; some of the data were only available for 2020. While socioeconomic data obtained from the CDC largely consists of ischemic heart disease along with other types of heart disease, it may be less generalizable to CMR due to the lack of more granular data. Third, one institution may have several affiliate CMR centers with each owning multiple magnets that are located in different zip codes (e.g. Los Angeles), which is represented in aggregate as institution-specific statistics in the survey data. Fourth, we assumed survey data are both accurate and representative of all US CMR centers. While the survey data are self-reported and only captured information from 112 centers, an effort was made to ensure that responses were from unique institutions. The survey was susceptible to response bias, as some responders did not complete the survey in its entirety. However, each question had a >90% response rate, except for the indications section. This was addressed by analyzing the proportion of responses within each indication rather than comparing sums of responses. No incentive was provided for participation in the survey, and results were not independently validated, which may also contribute to response bias. Small volume centers were not as well represented in both the survey data and CMS data. Survey data were also more reflective of centers with physicians active in the SCMR. Contact information was not available online for CMR centers without physician members in the SCMR and the exact number of scans was not available for centers that performed less than 10 scans per billing code. A higher proportion of respondents to the survey were cardiologists compared to radiologists and thus the answers to subjective questions, such as satisfaction with institution-specific factors, may reflect the views of respondents with greater representation and participation in the survey. Moreover, our survey did not capture the role that referral patterns play in the utilization of CMR and it also did not address the role that educational outreach may affect downstream CMR use. Both merit further study. Finally, the administrative report only included data on CMR billing practices for Medicare beneficiaries, while the survey captures data from imaging centers that provide CMR services to everyone, including those who are privately insured or uninsured.

## 6 Conclusions

Our findings further support geographic variability in CMR access. Future work could consider reporting distance-to-nearest CMR centers based on urban vs rural zip codes. Despite increasing volume and availability in almost all 50 states, CMR access remains geographically variable and underutilized. Center-specific barriers, such as disparate referral base, wait time, cost, CMR exam duration, protocol complexity, and experience, are potential contributors to the underutilization of CMR even when geographically accessible. Future research may be directed toward patient-related factors rather than physician- or facility-related barriers to CMR use. Such efforts may capture unique patient perspectives about barriers to CMR access and use.

## Ethics approval and consent

The study was exempt from Institutional Board Review.

## Funding

Dr. Nguyen receives grant support from the 10.13039/100000002National Institutes of Health (R01HL148182, R01HL1271533, R01HL169695) and the Veterans Health Administration (CX001901). Dr. Finn receives grant support from the 10.13039/100000002National Institutes of Health (R01HL148182, R01HL1271533, R01HL169695). The views published are those of the authors and do not reflect those of the UC Regents or the United States government.

## Author contributions

**Nazia Husain:** Writing – review and editing, Methodology, Data curation. **Robert W. Biederman:** Writing – review and editing, Methodology, Conceptualization. **J. Paul Finn:** Writing – review and editing, Supervision, Methodology, Conceptualization. **Anthon R. Fuisz:** Writing – review and editing, Supervision, Methodology, Data curation, Conceptualization. **Ibrahim M. Saeed:** Writing – review and editing, Supervision, Methodology, Data curation, Conceptualization. **Kim-Lien Nguyen:** Writing – review and editing, Visualization, Validation, Supervision, Resources, Project administration, Methodology, Investigation, Formal analysis, Data curation, Conceptualization. **Jennifer M. Li:** Writing – review and editing, Writing – original draft, Visualization, Validation, Methodology, Investigation, Formal analysis, Data curation, Conceptualization. **David R. Ho:** Writing – original draft, Methodology, Investigation, Formal analysis, Data curation.

## Consent for publication

Not applicable.

## Declaration of Generative AI and AI-assisted technologies in the writing process

Not applicable.

## Declaration of competing interests

The authors declare that they have no known competing financial interests or personal relationships that could have appeared to influence the work reported in this paper.
