# Supplementary material for: Regional variability of cardiovascular magnetic resonance access and utilization in the United States
Source: J Cardiovasc Magn Reson. 2024 Jul 11;26(2):101061. doi: 10.1016/j.jocmr.2024.101061 (PMC11663765; doi:10.1016/j.jocmr.2024.101061)
Supplement: Supplementary file 1 — Supplementary material [file mmc1.docx]

**SUPPLEMENTAL MATERIAL**

Table S1. Survey questions

| **Characteristics** | |
| --- | --- |
| - Please select which designations you hold (cardiologist, radiologist) - What year did your center start CMR? | |
| **Availability** | **Utilization** |
| - On which of the following scanners do you perform CMR? Please select all manufacturers and field strengths that you use. - What is/are the approximate age of your scanner(s) on which you perform CMR? - In what zip code are the scanners located? - What are the operating days of your scanner(s)? - What is the approximate wait time for a CMR scan at your center? - Do you have a program in place to perform MRIs in the setting of MR-conditional CIEDs? Non MR-conditional CIEDs? - When you do a congenital study, how often do you bill for both a cardiac and a thoracic MRA? - Are there CMR trainees in your department? - How many level II or III trained physicians currently read studies at your center? - Did the majority of your technologists receive training at a SCMR event? - Who reads these vascular studies? - For cardiology, how often do you use an over-read service from radiology? - How often do you perform combined reads from both services for cardiac studies? - How often do you perform combined reads from both services for vascular studies? - Do you feel supported by your institution to read CMRs from a time perspective? - Do you feel supported by your institution to read CMRs from a salary perspective? - Cardiology - How are you and your colleagues paid? - Radiology - How are you and your colleagues paid? - Do you split RVUs with radiology or cardiology? - Who processes your studies? (approximate percent of time - answers should ideally add to 100%)   - Me   - Fellow trainee   - Technologist   - AI - How do you report your studies? | - How many clinical CMR scans did you perform in 2017? 2018? 2019? - Approximately what percentage of your CMRs are performed at:   - Community   - Outpatient Imaging Center   - Academic Healthcare System - Approximately what percentage of your CMRs are performed:   - As an inpatient   - As an outpatient - Approximately what percentage of indications for your studies are the following:   - Evaluate cardiomyopathy   - Evaluate function and viability   - Stress perfusion MR   - Valve disease   - Pericardial disease   - Congenital heart disease   - Pulmonary hypertension   - Masses   - Acquired vascular disease - Approximately what percentage of your congenital heart disease studies are performed on:   - Pediatric patients   - Adults with congenital heart disease - What percentage of your pediatric patients receive some sort of sedation? - Do you do your Qp:Qs with: (net flows, forward flows)? - Approximately what percentage of your vascular cases are:   - Carotid   - Thoracic   - Abdominopelvic - Do you have a program in place to triage requests for CMR? - In approximately what percentage of your studies do you:   - Use 4D flow?   - Clinically assess and report with T1 mapping? T2 mapping?   - Clinically use strain imaging? - How often do you clinically quantify/semi-quantify myocardial blood flow on your stress perfusion? |
